# A highly sensitive Anti-Müllerian hormone test as a promising tool for follicle growth prediction in primary ovarian insufficiency patients

**DOI:** 10.1038/s41598-025-98808-0

**Published:** 2025-04-23

**Authors:** Zijia Guo, Bunpei Ishizuka, Atsuo Itakura, Kazuhiro Kawamura

**Affiliations:** 1 Department of Obstetrics and Gynecology, Juntendo University Graduate School of Medicine, 2-chome-1-1 Hongo, Bunkyo City, Tokyo, 113-8421 Japan; 2 Rose Ladies Clinic, 2-chome-3-18, Todoroki, Setagaya City, Tokyo, 158-0082 Japan; 3 Department of Obstetrics and Gynecology, Faculty of Medicine, Juntendo University, 2-chome-1-1 Hongo, Bunkyo City, Tokyo, 113-8421 Japan

**Keywords:** Primary ovarian insufficiency, Follicular development, Anti-Müllerian hormone, Predictive markers, Developmental biology, Endocrinology

## Abstract

Primary ovarian insufficiency (POI) patients often require prolonged stimulation for follicular growth. Anti-Müllerian hormone (AMH), produced by granulosa cells of early-stage follicles, is a potential a biomarker for predicting follicular development in POI patients undergoing ovarian stimulation. This retrospective study analyzed 165 patients undergoing 504 long controlled ovarian stimulation cycles. AMH levels were measured three weeks after stimulation initiation using a highly sensitive assay to guide decisions on extending stimulation beyond four weeks. Follicular development occurred in 9.7% of cycles among 41 patients, who had shorter amenorrhea durations and lower baseline follicle-stimulating hormone levels. Three-week AMH levels showed superior predictive ability for follicular development (area under the curve: 0.957; optimal threshold: 2.45 pg/ml) and were negatively correlated with time to follicular detection (R = − 0.326, P < 0.05). However, AMH levels did not significantly affect the precise time required for follicular development or show significant differences in oocyte yield or embryo quality. The study concludes that three-week AMH levels can predict follicular growth in POI patients. These findings suggest that a highly sensitive AMH assay could be a valuable tool for guiding ovarian stimulation in POI patients, potentially improving treatment outcomes.

## Introduction

Primary ovarian insufficiency (POI) is characterized by the early depletion of primordial follicles before age 40^1,2^, resulting in anovulation and amenorrhea^3^. While effective treatments to restore ovarian function lacking, POI can exhibit sporadic remission with intermittent follicular growth^4^, offering some hope for fertility. However, ovulation induction yields only a 6.3% pregnancy rate^3^. Despite recent advances, including in vitro activation (IVA) and drug-free IVA, which have shown promising results^5–8^, these techniques are still considered experimental, making oocyte donation the primary option. Ovulation induction typically takes about two weeks in women with normal ovarian reserve; however, development of follicles from the preantral to preovulatory stage requires more than two weeks. Therefore, we developed a prolonged controlled ovarian stimulation (COS) protocol lasting over four weeks for POI patients. COS was terminated at four week based on the absence of small antral follicles detected by ultrasound: if no follicles were observed , a new cycle was initiated. Our previous study demonstrated the efficacy of this protocol, increasing clinical pregnancy rates to 14.7%^9^. Ultrasound detects only follicles larger than 2 mm, potentially missing early-stage growth. Extending stimulation for cycles with early-stage follicles may lead to detectable growth, while for those without, it increases cost and health risks. Therefore, a sensitive biomarker is needed to detect early-stage follicle development, enabling better prediction of follicular growth and more effective treatment strategies for POI patients undergoing COS.

Estradiol (E2) is first produced by granulosa cells in late preantral follicles^10^. As follicles develop, the increasing number of granulosa cells, along with enhanced aromatase activity and androgen synthesis, contributes to a gradual rise in E2 levels^11^. While E2 is commonly used to predict follicular development, it has limitations in POI patients, often remaining undetectable and being influenced by exogenous estrogen^12^. Follicle-stimulating hormone (FSH) levels can predict oocyte yield in POI patients^13,14^, but are also affected by hormone therapy. This necessitates a more reliable biomarker than E2, FSH or ultrasound. Anti-Müllerian hormone (AMH), produced by granulosa cells, is minimally expressed in primordial follicles, low in primary follicles, and high in secondary, preantral, and small antral follicles up to 4 mm in diameter^15^. Its levels decrease as follicles mature^16^ and are absent in dominant follicles^17^. AMH reflects the continuous growth of small ovarian follicles, maintaining stable levels throughout the menstrual cycle^15,18^. Unlike E2 and FSH, AMH levels are unaffected by exogenous hormone treatments^19^, making it a reliable marker for developing follicular pools. In POI patients, AMH levels are typically very low due to the scarcity of growing follicles, often below the detection limit of common clinical assays.

The pico AMH ELISA (MenoCheck pico AMH, Ansh Labs) is a highly sensitive assay with a limit of detection (LoD) of 1.3 pg/mL^20^, significantly lower than commonly used AMH assays (Access AMH immunoassay: 0.02 ng/mL; Gen II AMH ELISA: 0.08 ng/mL). If follicles develop to the secondary stage, AMH levels should rise, but previous assays could not detect, such minimal increases. The pico AMH ELISA’s improved sensitivity allows for the detection of subtle AMH fluctuations in POI patients. Therefore, we hypothesized that very low AMH levels could predict follicular development in POI patients. This study retrospectively analyzed whether these very low AMH levels in POI patients undergoing COS can predict antral follicle development detected by ultrasound during the cycle and correlate with the timing of this development.

## Materials and methods

### Patients

We analyzed the clinical outcomes of COS cycles in POI patients treated at Rose Ladies Clinic in Tokyo, from February 2022 to May 2024. This study was approved by Juntendo University’s Research Ethics Committee (E23-0395). The study did not involve any experimental interventions or direct interaction with participants, as it was based solely on a review of existing medical records. For this reason, the requirement for individual informed consent was waived by the Ethics Committee. Instead, an opt-out approach was adopted to ensure participants were informed of the study and had the opportunity to decline participation. Details about the study, including its purpose and procedures, were disclosed on the website of Rose Ladies Clinic. The disclosure document used for this purpose was reviewed and approved by the Ethics Committee. All research was conducted in accordance with relevant guidelines and regulations, including the Declaration of Helsinki.

Inclusion criteria: 1) Age 20–48 years, seeking conception through COS-IVF or oocyte cryopreservation, 2) Last menstruation before age 40, with serum FSH > 25 mIU/mL and E2 < 20 pg/mL in at least two tests 4 weeks apart, and > 3 months amenorrhea without hormone therapy^2^, and 3) Serum AMH measured after 3 weeks of COS (days 18–27).

Exclusion criterion: Incomplete treatment cycles due to patient-related factors (e.g., fever, cold, or patient preferences to discontinue treatment).

### COS protocol

As described previously^9^, GnRH-α (Buserelin acetate) was administered on days 3–5 of withdrawal bleeding, which was induced by medroxyprogesterone acetate, after confirming normal serum FSH and LH levels (< 11 mIU/mL). For stimulation, human menopausal gonadotrophin or recombinant FSH were used. When a dominant follicle of 14–18 mm was present, stimulation was stopped, and 10,000–25,000 IU of human chorionic gonadotropin (hCG) was injected. Oocyte retrieval (OR) occurred 34–36 h post-hCG injection. If ovulation was observed before or during retrieval, intrauterine insemination (IUI) or timed intercourse was performed. If no follicular growth occurred after more than four weeks, ovarian stimulation was terminated, and medroxyprogesterone acetate was administered to induce withdrawal bleeding for the next cycle. In some cases, clomiphene citrate, or GnRH antagonist were administered based on patient characteristics. During the COS cycle, conjugated estrogen was administered orally for a priming treatment.

### Oocyte cryopreservation, in vitro fertilization (IVF), and IUI

For unmarried patients, metaphase II (MII) oocytes were vitrified within 2 h using the Cryotop vitrification kit (Kitazato Corp., Tokyo, Japan). For married patients, oocytes were cultured until IVF or intracytoplasmic sperm injection (ICSI) was performed as described previously^9^. Semen samples were analyzed according to WHO criteria^21^. Oocytes were inseminated or injected 3–5 h after OR. Fertilized oocytes were cultured for 24 h and graded using Veeck’s criteria^22^. High-quality embryos were defined as those at the four-cell stage with < 20% fragmentation. If embryos did not meet these criteria on day 2, culture was extended to day 5, and blastocyst were graded using Gardner’s criteria^23^. High and moderate-quality blastocysts were cryopreserved^24^. Due to suboptimal endometrial conditions from long-term COS, embryos were typically cryopreserved for future transfer. For IUI conversions due to early ovulation, prepared sperm suspension was transferred into the uterine cavity using a standard catheter.

### Hormone measurement and ultrasonography

Before COS, baseline data were collected, including FSH, LH, E2, and progesterone (P) levels on days 2–4 of withdrawal bleeding (or randomly for amenorrheic condition), baseline AMH levels, and ultrasound-detectable follicles count. Most patients underwent Access AMH immunoassay (Beckman Coulter Inc., Brea, CA) or Gen II AMH ELISA (Beckman Coulter Inc.), with some undergoing pico AMH ELISA staring February 2022. During COS, weekly serum measurements of FSH, LH, E2, and P were taken. At three weeks after COS initiation (days 18–27), serum AMH levels were assessed using pico AMH ELISA. Three time windows were defined: days 18–24, 21–27 and day 21, ensuring results, requiring one week to process, were available in the fourth week for clinical decisions regarding stimulation extension. After detecting ultrasound-visible antral follicles, hormonal and ultrasound monitoring increased until OR^25^.

Serum FSH, LH, E2, and P levels were quantified using an AIA-900 automated immunoassay analyzer (TOSOH AIA, Inc., Toyama, Japan). AMH was measured using either Access AMH immunoassay: intra- and inter-assay coefficients of variation (CV) 0.7–2.2% and 0.5%–1.4%; LoD 0.02 ng/mL, Gen II AMH ELISA: intra- and inter-assay CV 12.3% and 14.2%; LoD 0.08 ng/mL, or Pico AMH ELISA^26^: intra- and inter-assay CV 2.5–5.5% and 3.7–8.1%; LoD 1.3 pg/mL. Ultrasound-detectable follicle counts were defined as antral follicles (≥ 2 mm) detected via transvaginal or transabdominal ultrasonography, conducted by six physicians following standard procedures.

### Outcomes

The primary outcome of this study was to evaluate the predictive value of the 3-week AMH level, measured by pico AMH ELISA, for follicular development in the current treatment cycle using receiver operating characteristic (ROC) curve analysis. Successful follicular development was defined by ultrasonically detectable antral follicles. The ROC curve assessed sensitivity and specificity, with the area under the curve (AUC) indicating predictive accuracy.

Secondary outcomes included the correlation between 3-week AMH levels and follicular development timing (duration from the 3-week AMH measurement to ultrasound-visible follicles) and differences in AMH levels among groups with varying numbers of retrieved oocytes, mature oocytes, fertilized oocytes, and high-quality embryos.

### Statistical analysis

Data were analyzed using IBM SPSS Statistics for (version 26.0, SPSS Inc., Chicago, IL). Non-normally distributed continuous variables are reported as medians (interquartile range [IQR]) and compared using nonparametric tests. Categorical variables were presented as frequencies and percentages, compared using Fisher’s exact test. Predictive analysis utilized binary logistic regression and ROC curves, with the optimal AMH cut-off determined by maximizing the Youden index. Spearman’s rank correlation and linear regression assessed relationships between variables. Significance was set at *P* < 0.05.

## Results

### Baseline characteristics of enrolled patients and comparison of laboratory parameters between cycles with and without follicular growth

**Fig. 1:**
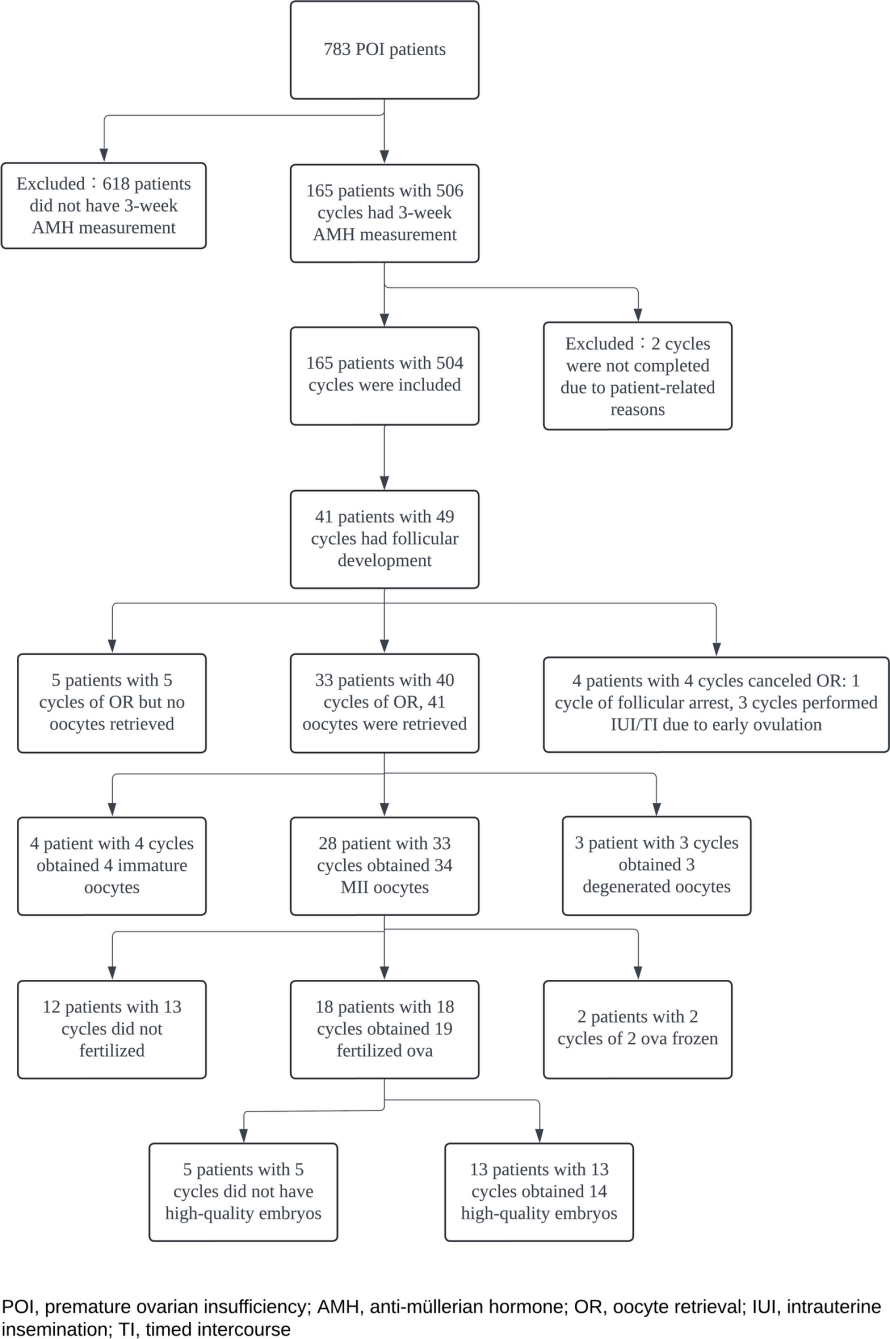
Flowchart of patient inclusion and outcomes in the study cohort. The flowchart illustrates the selection process of 783 patients with primary ovarian insufficiency (POI) and the subsequent analysis of 506 cycles. A total of 165 patients with 504 cycles were included after excluding patients without 3-week AMH measurements and incomplete cycles.Among these, 41 patients with 49 cycles exhibited follicular development. Subsequently, 33 patients with 40 cycles successfully retrieved 41 oocytes. Of these, 28 patients with 33 cycles obtained 34 mature oocytes (MII stage). Fertilization occurred in 18 patients across 18 cycles, resulting in 19 fertilized oocytes. Finally, 13 patients with 13 cycles developed 14 high-quality embryos.

To explore potential predictors of follicular development in POI patients, we evaluate baseline characteristics and hormonal profiles. As shown in Fig. 1, 165 POI patients underwent 3-week AMH measurements over 506 cycles, with 504 cycles included after excluding two incomplete cycles. Follicular growth was observed in 25% of patients (41/165).

As shown in Table 1, patients with follicular development exhibited significantly shorter amenorrhea duration, higher presumed age of onset, and higher BMI compared to those without follicular development. Other factors were similar between groups. Initially, patients with follicular development showed lower baseline FSH and more ultrasound-detectable follicles. However, analysis of 92 patients with recent data (within 1 year) revealed comparable LH, FSH and follicle numbers between groups: LH (median [IQR]): 31.5 [23.6, 39.5] vs. 34.05 [27.6, 44.55] mIU/mL, FSH: 86.1 [59.4, 102.6] vs. 88.25 [64.4, 113.38] mIU/mL, and follicle numbers: 0 [0, 1] vs. 0 [0, 1].

**Table 1:** Comparison of clinical and hormonal parameters between patients with and without follicle growth in 165 patients with primary ovarian insufficiency (POI).

Clinical parameters	Total (n = 165)	Without follicle growth (n = 124)	Follicle growth (n = 41)	*P*-value^a^
Female age (years)	36 (32, 41)	36 (32, 41)	38 (33, 41)	0.133^b^
Presumed age of POI onset (years)	30 (24, 37)	29 (23, 35)	34 (27, 38)	0.004^b^
Duration of amenorrhea (years)	5 (3, 9.75)	6.5 (3, 11)	4 (2, 6)	0.003^b^
BMI (kg/m^2^)	20.67 (19.15, 22.57)	20.59 (19.15, 22.36)	21.34 (20.03, 22.94)	0.048^b^
Baseline LH (mIU/mL)	33.1 (23.2, 43.3)	33.35 (22.25, 43.3)	30.6 (21.3, 36)	0.065^b^
Baseline FSH (mIU/mL)	78.1 (60.32, 102.45)	84.8 (60.60, 109.68)	73.7 (50.2, 98.7)	0.045^b^
Baseline ultrasound-detectable follicle number	0 (0, 0.75)	0 (0, 0)	0 (0, 1)	0.018^b^
Clinical pregnancy history	17/163 (10.43%)	11/123 (8.94%)	6/40 (15%)	0.21^c^
Chromosomal abnormality	15/148 (10.14%)	10/112 (8.93%)	5/36 (13.89%)	0.284^c^
Elevated serum autoantibody	66/160 (41.25%)	52/119 (43.7%)	14/41 (34.15%)	0.188^c^

BMI, body mass index; LH, luteinizing hormone; FSH, follicle-stimulating hormone.

Values for continuous variables are presented as median (25th, 75th percentile), and categorical variables as frequency and percentage (n/N, %).

^a^Compare without follicle growth and follicle growth groups.

^b^Mann–Whitney U test.

^c^Fisher exact test.

Baseline AMH was measured for 165 patients: Access AMH immunoassay or Gen II AMH ELISA (133 patients): 74% (99/133) had AMH levels below the LoD. Thirty-four patients had measurable levels (0.02–0.19 ng/mL), and only 7 of these were measured within 1 year before the cycle start (median 0.03 ng/mL, IQR: 0.025–0.055 ng/mL); Pico AMH ELISA (32 patients): 14 had levels below the LoD, and 18 ranged from 1.4 to 17.4 pg/mL. Most of these patients were diagnosed with POI more than one year prior, with undetectable AMH levels. However, due to multiple COS cycles with short intervals between cycles, AMH levels could not be measured before the start of every cycle.

**Fig. 2:**
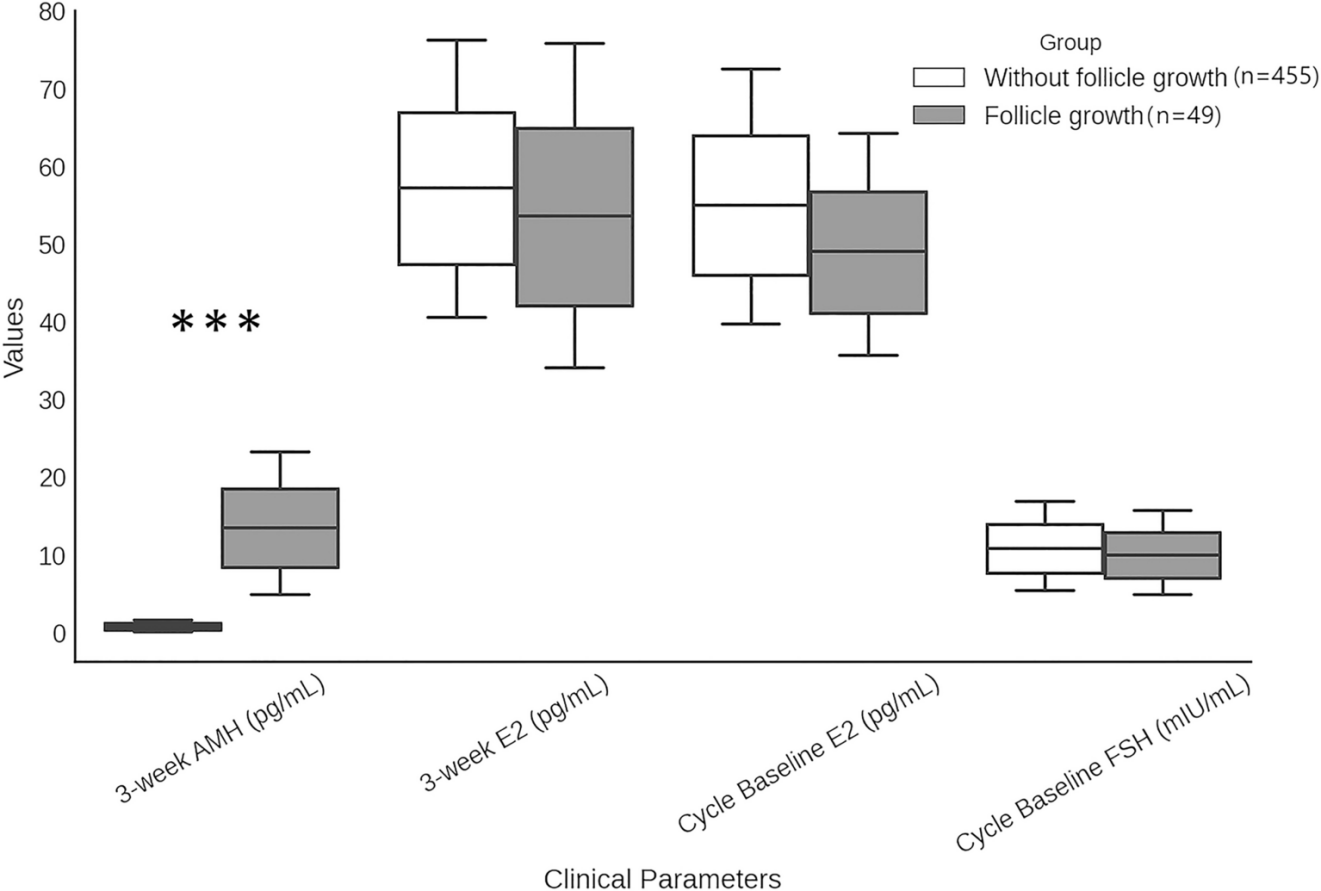
Cycle-based analysis of clinical and hormonal parameters in cycles with and without follicle growth. Box plots illustrate the distribution of 3-week Anti-Müllerian Hormone (AMH), 3-week estradiol (E2), cycle baseline E2, and cycle baseline follicle-stimulating hormone (FSH) levels across groups with and without follicular growth. The plots dispict the median, interquartile range (25th–75th percentile), and overall data distribution for each hormonal parameter. Statistical comparisons were performed using the Mann–Whitney U test. ***P < 0.001.

Among analyzed cycles (Fig. 2), follicular growth occurred in 9.72% (49/504), with significantly higher 3-week AMH levels in the follicular growth group (median 7.0 pg/mL, IQR: 4.78–23.45 pg/mL) compared to the no follicular growth group (median 0.0 pg/mL, IQR: 0.0–1.6 pg/mL). No significant differences were found in 3-week E2 (55.25 pg/mL vs. 59.0 pg/mL, P = 0.634), cycle baseline E2 (48.25 pg/mL vs. 54.35 pg/mL, *P* = 0.243), or cycle baseline FSH levels (9.15 mIU/mL vs. 9.1 mIU/mL, *P* = 0.658) between groups.

Bivariate logistic regression analysis of significant factors from Tables 1 and Fig. 2 revealed that only the 3-week AMH level significantly predicted follicular growth (Table 2).

**Table 2:** Bivariate logistic regression analysis of clinical parameters impacting follicular growth in patients with primary ovarian insufficiency (POI).

Clinical parameters	*P*-value	Odds ratio	95% C.I.for odds ratio	
			Lower	Upper
3-weekAMH	0.000	1.339	1.213	1.479
Duration of amenorrhea	0.821	1.017	0.879	1.176
Presumed age of POI onset	0.100	1.076	0.986	1.175
BMI	0.946	1.005	0.866	1.167
Baseline FSH	0.474	0.995	0.981	1.009
Baseline ultrasound-detectable follicle number	0.101	1.782	0.893	3.556

AMH, anti-müllerian hormone; BMI, body mass index; FSH, follicle-stimulating hormone.

### The 3-week AMH levels emerge as key predictor of follicular growth in POI patients

**Fig. 3:**
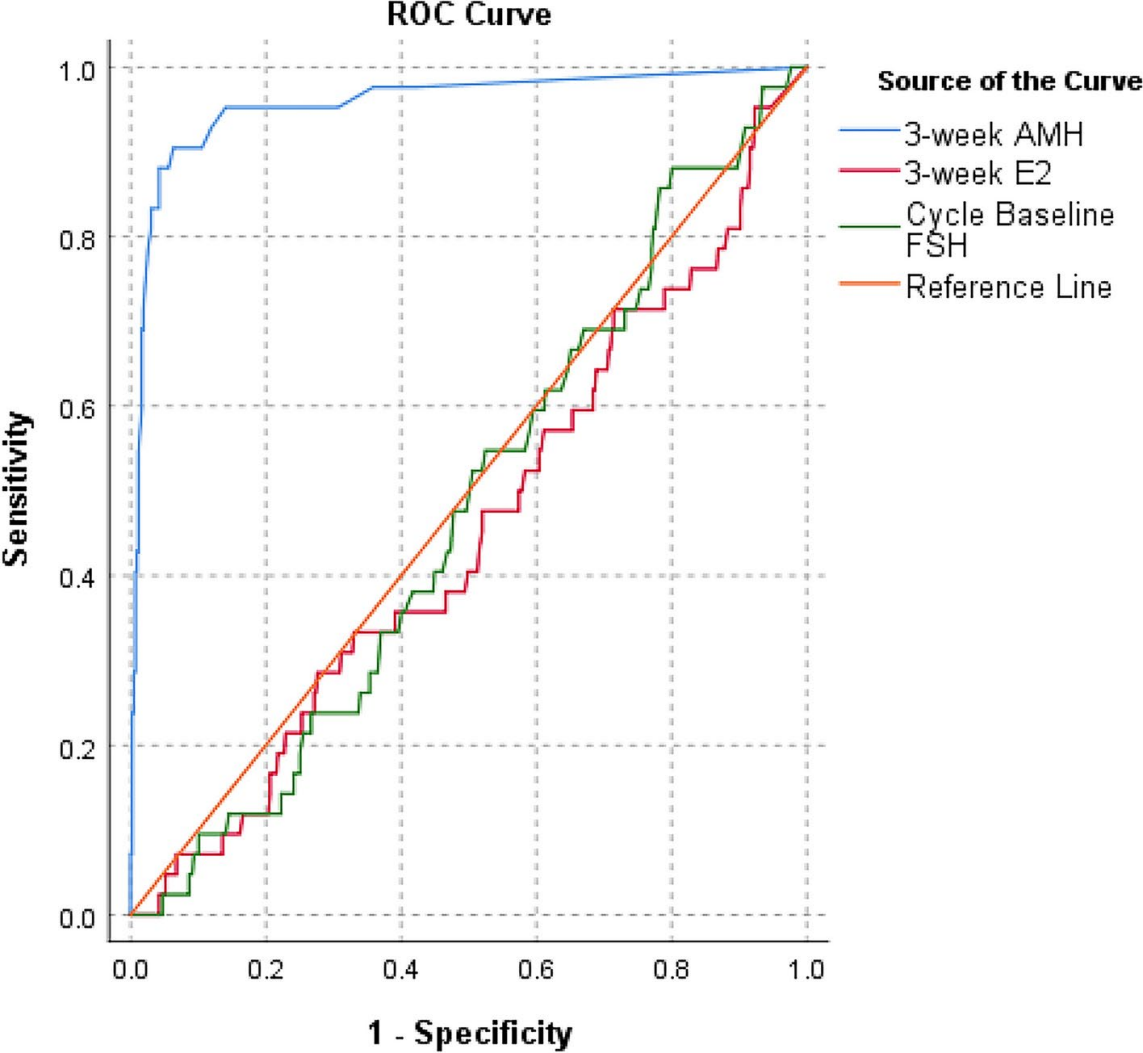
Receiver Operating Characteristic (ROC) curves for the prediction of follicular development. ROC curves display the diagnostic performance of four biomarkers: 3-week Anti-Müllerian Hormone (AMH), 3-week estradiol (E2), and cycle baseline follicle-stimulating hormone (FSH). Sensitivity is plotted against 1-specificity for each predictor. The area under the curve (AUC) values are as follows: 3-week AMH demonstrates superior performance with an AUC of 0.957 (95% Confidence Interval (CI): 0.920–0.995, *P* < 0.01), indicating excellent predictive accuracy for follicular development. In contrast, 3-week E2 and Cycle Baseline FSH have limited predictive value, with AUCs of 0.453 (95% CI: 0.361–0.545, *P* > 0.05) and 0.479 (95% CI: 0.395–0.564, *P* > 0.05), respectively. The reference line represents a non-informative test.

To identify effective biomarker for predicting follicular development in POI patients, we compared the predictive capabilities of 3-week AMH, 3-week E2, and cycle baseline FSH. ROC curve analysis showed that 3-week AMH had strong predictive capability for follicular development, with an AUC of 0.957 (95% CI: 0.920–0.995, *P* < 0.01). In contrast, 3-week E2 and cycle baseline FSH were weak predictors (Fig. 3).

We assessed AMH’s predictive performance across three measurement timing groups: days 18–24, days 21–27, and day 21 (Table 3). All groups had an optimal cut-off value of 2.45 pg/mL, but the day 21 group exhibited the highest sensitivity and specificity, indicating superior accuracy in predicting ovarian response. The AUC for the day 21 measurement was higher than for those taken on days 18 to 24 and days 21 to 27, with the narrowest 95% CI. Therefore, scheduling AMH measurements on day 21 of the stimulation cycle may yield the most informative results regarding potential follicular development.

**Table 3:** Optimal cut-off values for pico AMH ELISA across different cycle days.

Measurement group	Number of cycles/ Number of patients	AUC	*P*-value	Optimal cut-off (pg/mL)	Sensitivity	Specificity	Asymptotic 95% CI	
							Lower	Upper
3-weekAMH (days 18–24)	473/159	0.957	0.000	2.45	0.909	0.935	0.921	0.993
3-weekAMH (days 21–27)	392/149	0.956	0.000	2.45	0.912	0.947	0.911	1.001
3-weekAMH (day 21)	271/117	0.986	0.000	2.45	0.957	0.960	0.974	0.999

AMH, anti-müllerian hormone.

AUC = area under the curve; CI, confidence interval.

Days 18–24, days 21–27, and days 21: Samples obtained from day 18 to day 24, day 21 to day 27, and day 21 of the stimulation cycle, respectively.

### AMH levels correlate with follicular development time but limited predictive power

We hypothesized that higher AMH levels could be associated with shorter follicular development times. To test this, we performed a Spearman correlation analysis between 3-week AMH levels and the time required for follicles to become ultrasound-detectable. Cycles with follicular development averaged 22.06 days after the 3-week AMH ELISA (median 15 days, IQR: 12–25 days). A significant negative correlation between 3-week AMH levels and follicular development time (Table 4). However, linear regression analysis showed that even after log transformation, AMH levels did not significantly predict development time. These results suggest that while higher AMH levels correlated with shorter development times, this relationship is not strong enough to reliably predict follicular development duration.

**Table 4:** Spearman correlation and linear regression analysis between 3-week AMH levels and time required for follicle development.

Analysis type	Correlation coefficient (R) / unstandardized coefficient (B)	Std. error	t-value	*P*-value
Spearman correlation (R)	− 0.326	–	–	0.022
Linear regression (B)	− 0.072	0.184	− 0.393	0.696
Linear regression (log-transformed B)	− 0.169	0.114	− 1.486	0.144

AMH, anti-müllerian hormone.

Time required for follicle development = the duration from the 3-week AMH assay to the appearance of ultrasound-visible follicles.

### The 3-week AMH levels lacked the association with oocyte yield and embryo quality in POI patients

**Fig. 4:**
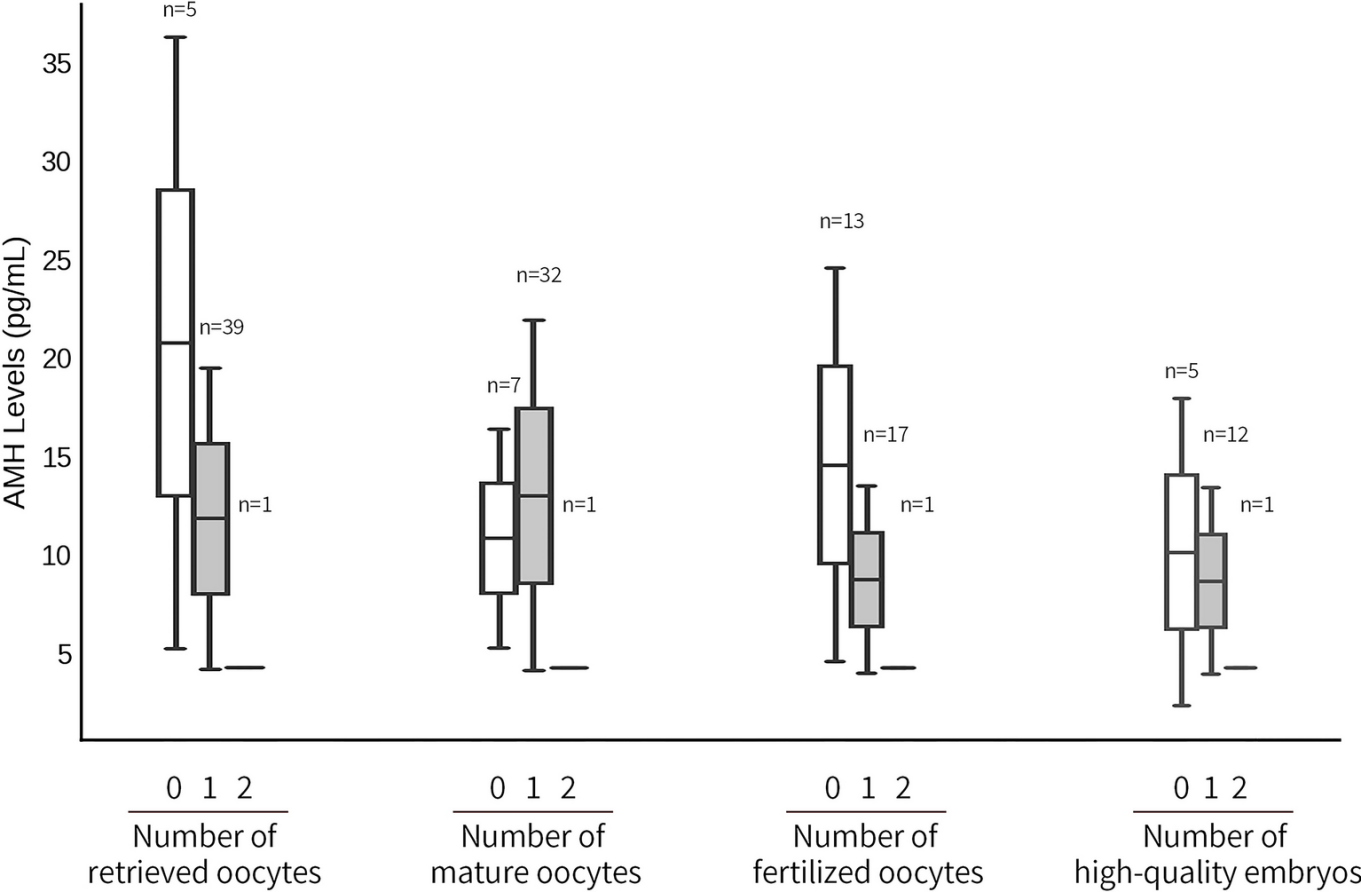
Relationship between 3-week AMH levels and IVF outcomes. Box plots illustrate the distribution of 3-week Anti-Müllerian Hormone (AMH) levels across groups categorized by the number of retrieved oocytes, mature oocytes (MII), fertilized oocytes, and high-quality embryos. AMH levels are presented as medians with interquartile ranges. No cycle yielded more than two oocytes. Statistical comparisons were performed using the Kruskal–Wallis H test, revealing no significant differences in AMH levels among the groups (*P* > 0.05).

We examined the relationship between 3-week AMH levels measured by pico AMH ELISA and key IVF outcomes in POI patients. AMH levels were compared across groups based on the number of retrieved oocytes, mature oocytes, fertilized oocytes, and high-quality embryos, noting that no cycle yielded more than two oocytes. Kruskal–Wallis H tests revealed no significant differences in AMH levels among any of the groups (Fig. 4). These findings suggest that in POI patients, 3-week AMH levels are not associated with oocyte yield and embryo quality.

## Discussion

A highly sensitive 3-week AMH level with a threshold of 2.45 pg/mL was identified as a superior predictor of follicular growth in the current stimulation cycle. While 3-week AMH levels negatively correlated with follicular development time, they could not reliably predict the specific duration. Additionally, 3-week AMH levels showed no association with oocyte yield or embryo quality in POI patients.

A previous study established AMH as a predictor of ovarian response in the general IVF population, primarily in terms of cycle cancellation^27^. This predictive potential has also been observed in POI patients, as demonstrated by Nagoya University study using pico AMH ELISA reported similar findings^13^, measuring AMH during the withdrawal bleeding phase to predict follicular growth in POI patients undergoing HRT. With a smaller sample size (19 patients, 91 cycles), they could not conduct ROC analysis for an optimal AMH threshold. However, their median AMH value of 2.77 pg/mL in the follicular development group closely aligns with our optimal cut-off, supporting the reliability of the high-sensitive AMH assay for predicting follicular development in POI patients.

The prolonged duration of ovarian stimulation in our study population is primarily due to the absence of antral follicles at the start of stimulation. In patients with normal ovarian reserve, COS begins with the pre-existing antral follicles, typically requiring 10–14 days to achieve follicular maturation. However, in our cohort of POI patients, there are no detectable antral follicles at the start of stimulation, necessitating COS to begin from an earlier stage of folliculogenesis. This earlier starting point requires a longer duration to achieve follicular growth and maturation. AMH exhibits an inverse relationship with the duration of follicular growth. However, this relationship is nonlinear; increases in AMH do not correspond to proportional decreases in development time. This may result from the varying contribution of small follicles at different stages to circulating AMH levels^28^. Multiple small follicles can release AMH comparable to a single larger follicle. While higher AMH levels are generally associated with shorter times for detecting ultrasound-visible follicles, delayed growth may suggest secretion from several smaller follicles, resulting in longer development times.

Regarding E2 and FSH, a previous study found that baseline E2 levels above 15.5 pg/mL predicted follicular development and ovulation in POI patients undergoing HRT^10^; however, exogenous estrogen complicates this assessment. Our study found no association between baseline E2 or 3-week E2 levels and follicular growth during stimulation. Due to consistent HRT, E2 levels were significantly affected by exogenous hormones, making E2 an unreliable indicator of follicular development. Additionally, AMH is produced by secondary to small antral follicles^15^ and can indicate early follicular development before significant estrogen production. Thus, AMH is a more sensitive and reliable predictor than E2 for POI patients undergoing COS.

Our findings regarding the relationship between FSH and follicular development differ from two studies. The Nagoya University study reported that lower cycle baseline FSH levels are associated with successful follicle growth, with a median level of 15.44 mIU/mL^13^. Another study found that ethinyl estradiol pretreatment improved ovulation induction success in POI patients when FSH levels were < 15 IU/mL before stimulation^29^. However, consistent with our results, a Jikei University study demonstrated no significant differences in FSH levels between cycles with and without follicular development^30^, reporting lower median FSH levels than the other studies^13,29^. The discrepancy may arise from the continuous estrogen use during the withdrawal bleeding phase in both our study and the Jikei University study, which could suppress FSH levels. Therefore, baseline FSH is not a reliable predictor of follicle growth in POI patients undergoing HRT.

In this study, the follicle growth rate per patient was 25%, substantially lower than the 48.3% reported in our previous study^9^. This discrepancy is primarily attributed to differences in patient populations, with the present study including individuals with more severe POI. In the present study, high-sensitivity AMH measurement at three weeks of ovarian stimulation was conducted exclusively in patients who exhibited no signs of follicular development by this timepoint. In contrast, the previous study^9^ included POI patients regardless of their follicular response at three weeks, incorporating individuals who had already demonstrated earlier follicular growth. Moreover, while most patients in both studies had baseline AMH levels below the detection limit, the maximum AMH in the previous study^9^ reached 0.65 ng/mL, whereas in the current study, it was only 0.19 ng/mL, indicating a more advanced stage of ovarian insufficiency. These differences in patient selection likely account for the lower follicular growth rate observed in the present study.

This study identified significant differences in baseline characteristics between patients with and without follicular development. Patients with follicular development had a shorter duration of amenorrhea, a higher presumed age of onset, a higher BMI, lower baseline FSH levels, and more ultrasound-detectable follicles. Our earlier study indicated that shorter amenorrhea duration correlates with better COS response in POI patients^31^. Patients with a later age of POI onset typically retain better reserves than those with an earlier onset at the same chronological age^32^. Although there is generally a negative correlation between BMI and AMH levels^33^, it is unclear why POI patients with follicular development showed a higher BMI in this study. Lower FSH levels and more detectable follicles indicate better ovarian function^34,35^; however, these factors do not reliably predict follicular development in the current cycle.

Ovarian reserve encompasses both the quantity and quality of follicles, with AMH recognized as an indirect marker^36^. While many studies link AMH to oocyte quantity^37–51^, its correlation with oocyte or embryo quality remains inconclusive^45,52–56^. Our study confirms that 3-week AMH can predict follicular development in POI patients, but does not substantiate its role in predicting oocyte quantity or embryo quality. The unique clinical presentation of POI makes follicular growth challenging; even patient with the highest follicle counts developed only two follicles. The pico AMH ELISA used offers high sensitivity for detecting minute variations in AMH levels. Although POI patients may have elevated AMH levels, these remain low compared to women with normal ovarian function. Under COS, retrieved oocytes are typically limited to one, complicating the assessment of oocyte quantity or embryo quality using 3-week AMH. Additionally, the small sample size may affect the validity of these assessments.

This study is the first to establish a precise threshold for extremely low AMH levels based on a large number of COS cycles as a predictor of follicular development in POI patients, providing a quantifiable benchmark for clinical application. However, limitations include variability in baseline data due to different assay methods for measuring AMH levels obtained from different hospitals. Additionally, the relationship between AMH levels and both oocyte quantity and embryo quality remain inconclusive, likely due to the small sample size.

## Conclusion

In conclusion, the 3-week AMH level can predict follicular growth in POI patients; however, does not predict the time required for follicle development, oocyte quantity, or embryo quality.

## Data Availability

The datasets generated during and/or analyzed during the current study are available from the corresponding author on reasonable request.
